# Adjusting perioperative methadone dose for elderly and fragile hip fracture patients (MetaHip-trial) – A statistical analysis plan for an adaptive dose-finding trial

**DOI:** 10.1016/j.conctc.2023.101228

**Published:** 2023-11-14

**Authors:** Andreas Kristian Pedersen, Kevin Heebøll Nygaard, Sofie Ronja Petersen, Kirsten Specht, Thomas Strøm, Caroline Margaret Moos, Helene Skjøt-Arkil, Jesper Ougaard Schønnemann

**Affiliations:** a Department of Clinical Research, University Hospital of Southern Denmark, Kresten Phillipsens vej, Aabenraa, Denmark; b Department of Orthopedics, University Hospital of Southern Denmark, Kresten Phillipsens vej, Aabenraa, Denmark; c Center for COPD, Center for Health and Rehabilitation, Randersgade 60, 2100, København Ø, Denmark; d Department of Anesthesiology and Intensive Care, University Hospital of Southern Denmark, Kresten Philipsens vej 15, 6200, Aabenraa, Denmark; e Emergency Department, University Hospital of Southern Denmark, Kresten Phillipsens vej, Aabenraa, Denmark

**Keywords:** Elderly, Fragile, Postoperative, Pain, Perioperative, Dose finding, Opioid consumption, CRM, Methadone

## Abstract

**Background:**

The elderly population is expanding globally. This gives numerous challenges especially regarding hip fracture patients. In the US alone over 300.000 hip fracture patients are treated each year, and a large amount of those develop opoid addiction. Hip fractures require surgical intervention within 24 h and is associated with significant pain even at rest. Postoperative analgesic treatment need to be optimized to ensure adequate pain relief and to prevent subsequent opioid addiction. Previous studies have shown that methadone effectively decreases post-operative opioid consumption but the studies focused on younger patients undergoing elective surgery. This study focus on the use of methadone on the elderly, fragile patients undergoing acute surgery, by first determining the maximal tolerable dose.

The hypothesis is the maximal tolerable doses of these hip-fracture patients lies between 0.10 mg/kg and 0.20 mg/kg. This trial aims to estimate the maximum tolerable dose of methadone when administered to elderly patients undergoing surgery for a hip fracture.

**Method:**

This project is an adaptive dose-finding trial. The continuous reassessment method will estimate the maximum tolerable dose of methadone. The primary outcome will be respiratory depression. The statistical analysis plan will be published a priori to the closure of patient recruitment and statistical analysis of database results.

**Conclusion:**

The results of this study will give valuable information about the maximally tolerated dose of methadone for postoperative pain relief for elderly patients with hip fractures and potential adverse events.

This trial is registered on clinicaltrials.gov with trial registration: NCT05581901. Registered 17 October 2022, https://www.clinicaltrials.gov/ct2/show/NCT05581901?term=methadone&cond = hip&draw = 2&rank = 1.

## 1 Introduction

### 1.1 Background and rationale

Patients with hip fractures represent a vast population with substantial societal costs [1,2]. Danish hospitals encounter 140 hip fracture patients per 100,000 inhabitants per year, approximately 8000 fractures annually [3]. The median age of hip fracture patients is 81 years indicating that the fragile and elderly are greatly affected. In Denmark the 1-year mortality in hip fracture patients is rougly 30 %, with approximately 10 % of previously self-reliant patients referred to nursing homes after discharge [3]. Furthermore, half of all hip fracture patients experience a permanent decrease in gait function [4].

Hip fractures are classified as collum femoris fractures (50 %), pertrochanteric fractures (40 %) and subtrochantheric fractures (10 %). They all require surgical interventions within 24 h and are associated with significant pain even at rest [5]. Studies have indicated that postoperative pain is insufficiently treated in more than half of hip fracture patients, increasing the risk of chronic pain, delaying discharge and preventing early mobilization and rehabilitation [6].

Numerous studies suggest, that a single dose of methadone given during the surgery significantly ameliorates postoperative pain and reduces postoperative opioid consumption [[7], [8], [9], [10]]. However, these studies investigated younger patients undergoing elective surgery. At Hospital Sønderjylland (SHS), perioperative methadone is used for isolated cases, e.g., patients with chronic pain or high morphine tolerance. However, methadone is not part of routine care and is not used for the elderly. Therefore exploring the maximal tolerable dose (MTD) for elderly patients is clinically relevant.

The treatment of postoperative pain has been improved by a multimodal approach and using peripheral nerve blocks. However, supplemental opioids are often necessary, commonly taken months or years after surgery, and are an increasing healthcare challenge [11]. Consequently, patients undergoing surgery are at risk of experiencing side-effects and developing physical as well as psychological addiction to opioids [12]. The most common opioid-related side-effects include obstipation, nausea, itchy skin, dry mouth, vertigo, and sedation [7,12]. Therefore, opportunities to decrease the need for opioids in the postoperative phase are highly relevant.

In most studies, methadone is given perioperatively in dosages of 0.10–0.30 mg/kg and commenced at the induction of anaesthesia or surgery [7,10,13,14]. However, studies investigating the optimal dosage and time for administration reported that the patients receiving methadone after surgery needed twice as much opioid pain medication at postoperative day one compared to patients receiving methadone at induction of anaesthesia [13]. These studies also show that when using smaller dosages, such as 5–10 mg, the analgesic duration was only 3–4 h, compared with doses of 20 mg or more, which have a clinical effect closely related to the elimination half-life of 15–60 h without any increased risk for respiratory depression [13]. However, it is unclear how these doses are tolerated in an elderly and fragile population, and therefore the maximal tolerable dose needs to be determined.

## 2 Aim

This trial aims to estimate the maximum tolerable dose of methadone when administered to patients *≥* 60 years undergoing surgery for a hip fracture.

## 3 Statistical hypothesis

We hypothesize that the maximal tolerable dose of methadone for patients with a hip fracture lies between 0.10 mg/kg and 0.20 mg/kg.

## 4 Objective

The trial estimates the maximal tolerable dose by investigating the risk of respiratory depression in the first 24 h after surgery for patients *≥* 60 years undergoing surgery for a hip fracture. The secondary objectives include how the methadone doses are tolerated in regard to side effects and consumption of rescue medication.

## 5 Study methods

### 5.1 Study design, setting and recruitment

A single-centre adaptive dose-finding trial will be conducted. The recruitment will, according to the plan, commence in November 2022. All hip fracture patients from the regional Hospital Sønderjylland orthopaedic or emergency department will be invited to participate. The on-duty orthopaedic personnel will recruit patients and ensure the patient receives written and verbal information about the study. The patients will be offered 2 h to consider participation.

### 5.2 Inclusion and exclusions criterias

#### 5.2.1 Inclusion criteria

• Patients presenting with a hip fracture in the emergency department (collum femoris fractures, pertrochanteric fractures and subtrochanteric fractures. ICD-10-codes: DS720-722)
• Age *≥* 60 years
• Patients must be able to reliably assess their pain level using the Verbal Rating Scale (VRS) and be able to ask for supplementary analgesics as needed
• Patients must be able to read and speak Danish and understand the information and be able to give informed consent

#### 5.2.2 Exclusion criteria

• Polytrauma (defined as severe injuries to multiple body regions) or multiple indications for surgical intervention (defined as one or more absolute indications for surgical intervention besides the hip fracture).
• Previous allergic reactions or hypersensitivity towards methadone hydrochloride or sodium-chloride.
• Contraindications for methadone treatment, i.e. chronic obstructive pulmonary disease (Gold classification C + D [15]), history with acute asthma attacks or atopic skin conditions, cor pulmonale, raised intracranial pressure or head injury, pheochromocytoma, history with paralytic ileus, QT interval prolongation, myasthenia gravis, liver disorders or hypotension.
• Concurrent administration with monoamin oxidase (MAO) inhibitors or within 2 weeks of suspending treatment with these medicinal products.
• Concurrent administration of sedatives, e.g. benzodiazepines or related drugs.
• Included in other studies.
• Current drug addiction, e.g., opioid addiction or intravenous addiction.

### 5.3 Study population characteristics

The variables will be presented in a baseline table (see Table 1). Descriptive statistics are presented below in section 7.1 and used to assess reproducibility of our data to other settings.

**Table 1:** Baseline table.

Variables	Methadone dose group			P-value
	0.10 mg/kg	0.15 mg/kg	0.20 mg/kg	
*Hip fracture type*				
Collum femoris fracture DS720	xx (xx,x%)	xx (xx,x%)	xx (xx,x%)	*△ or***┼**
Pertrochanteric fracture DS721	xx (xx,x%)	xx (xx,x%)	xx (xx,x%)	*△ or***┼**
Subtrochanteric fracture DS722	xx (xx,x%)	xx (xx,x%)	xx (xx,x%)	*△ or***┼**

*Demographic data*				
Age	xx ± xx	xx ± xx	xx ± xx	* *or ₼*
Sex	xx (xx,x%)	xx (xx,x%)	xx (xx,x%)	*△ or***┼**
ASA (American Society of Anesthesiologists) classification	xx (xx,x%)	xx (xx,x%)	xx (xx,x%)	*△ or***┼**

*Lifestyle*				
Weight (BMI)	xx ± xx	xx ± xx	xx ± xx	* *or ₼*
Tobacco	xx (xx,x%)	xx (xx,x%)	xx (xx,x%)	*△ or***┼**
Alcohol	xx (xx,x%)	xx (xx,x%)	xx (xx,x%)	*△ or***┼**
Chronic use of opioids	xx (xx,x%)	xx (xx,x%)	xx (xx,x%)	*△ or***┼**

*Comorbidities*				
Congestive and chronic heart failure	xx (xx,x%)	xx (xx,x%)	xx (xx,x%)	*△ or***┼**
Cardiac arrhythmias	xx (xx,x%)	xx (xx,x%)	xx (xx,x%)	*△ or***┼**
Valvular disease	xx (xx,x%)	xx (xx,x%)	xx (xx,x%)	*△ or***┼**
Pulmonary circulation disorders	xx (xx,x%)	xx (xx,x%)	xx (xx,x%)	*△ or***┼**
Peripheral vascular disorders	xx (xx,x%)	xx (xx,x%)	xx (xx,x%)	*△ or***┼**
Hypertension, uncomplicated	xx (xx,x%)	xx (xx,x%)	xx (xx,x%)	*△ or***┼**
Hypertension, complicated	xx (xx,x%)	xx (xx,x%)	xx (xx,x%)	*△ or***┼**
Paralysis	xx (xx,x%)	xx (xx,x%)	xx (xx,x%)	*△ or***┼**
Other neurological disorders	xx (xx,x%)	xx (xx,x%)	xx (xx,x%)	*△ or***┼**
Chronic pulmonary disease	xx (xx,x%)	xx (xx,x%)	xx (xx,x%)	*△ or***┼**
Diabetes, uncomplicated	xx (xx,x%)	xx (xx,x%)	xx (xx,x%)	*△ or***┼**
Diabetes, complicated	xx (xx,x%)	xx (xx,x%)	xx (xx,x%)	*△ or***┼**
Hypothyroidism	xx (xx,x%)	xx (xx,x%)	xx (xx,x%)	*△ or***┼**
Renal failure	xx (xx,x%)	xx (xx,x%)	xx (xx,x%)	*△ or***┼**
Liver disease	xx (xx,x%)	xx (xx,x%)	xx (xx,x%)	*△ or***┼**

Statistical test for the p-value.

*∗ Anova*.

**┼***χ*^*2*^.

*△ Fischer's exact*.

*₼ Kruskal-Wallis test*.

## 6 Variables

### 6.1 Baseline variables

For safety and reproducibility purposes, the baseline variables presented in Table 1 will be noted for each given methadone dose in the trial (see Table 2).

**Table 2:** Secondary analysis.

Secondary analysis					
Variable	Level			Coef (95%CI)	p-value
Length of stay Pain	0.10 mg/kg 0.15 mg/kg 0.20 mg/kg 0.10 mg/kg 0.15 mg/kg 0.20 mg/kg			Ref. Ref.	n.a n.a
Variable	Level			IRR (95%CI)	p-value
Consumption	0.10 mg/kg	at	6 h	Ref.	
	0.15 mg/kg	at	6 h		
	0.20 mg/kg	at	6 h		
	0.10 mg/kg	at	24 h	Ref.	
	0.15 mg/kg	at	24 h		
	0.20 mg/kg	at	24 h		
	0.10 mg/kg	at	72 h	Ref.	
	0.15 mg/kg	at	72 h		
	0.20 mg/kg	at	72 h		
Respiratory depression	0.10 mg/kg			Ref.	n.a
	0.15 mg/kg				
	0.20 mg/kg				
Variable	Level			OR (95%CI)	p-value
Side effects	0.10 mg/kg	at	6 h	Ref.	
	0.15 mg/kg	at	6 h		
	0.20 mg/kg	at	6 h		
	0.10 mg/kg	at	24 h	Ref.	
	0.15 mg/kg	at	24 h		
	0.20 mg/kg	at	24 h		
	0.10 mg/kg	at	72 h	Ref.	
	0.15 mg/kg	at	72 h		
	0.20 mg/kg	at	72 h		

### 6.2 Primary outcome

The primary outcome is the incidence of respiratory depression during the first 24 h after surgery. Respiratory depression is defined as a respiratory frequency below 10 breaths pr. minute and peripheral oxygen saturation below 94 % despite 4 ltr/min oxygen and will be registered upon arrival at the orthopaedic ward and again 6 and 24 h after surgery.

### 6.3 Secondary outcomes

The secondary outcomes include:

• Length of stay, defined as the number of hours spent at the PACU.
• Number of times administration of antidote Naloxone was necessary.
• Consumption of opioid rescue medication measured upon arrival at the orthopaedic ward and after 6, 24, and 72 h postoperatively.
• Opioid-related side effects, including post-operative nausea and vomiting (PONV), measured upon arrival at the orthopaedic ward and after 6 and 24 h postoperatively.
• Postoperative pain will be measured using VRS upon arrival at the orthopaedic ward and after 6, 24, and 72 h postoperatively.

The primary and secondary outcomes will be measured by trained healthcare personnel at the orthopaedic department and recorded in RedCap (Research electronic data Capture). In addition, KHN will note the starting dose and whether the patient experienced toxicity in the dose-escalation program.

### 6.4 Exposure

The exposure is a categorical variable where the groups are defined as:

• 0.10 mg/kg methadone at the beginning of surgery.
• 0.15 mg/kg methadone at the beginning of surgery.
• 0.20 mg/kg methadone at the beginning of surgery.

**Fig. 1:**
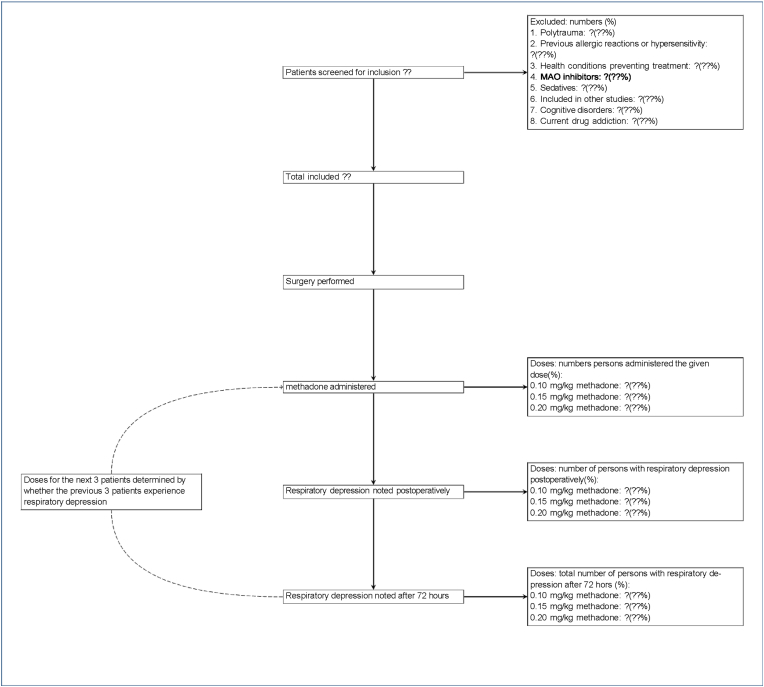
Flowchart and treatment allocation in the trial.

When patients are included from the ED the orthopaedic doctor will contact the primary investigator KHN, who will assign each patient to a methadone group (0,10 mg/kg, 0,15 mg/kg or 0,20 mg/kg) using the model-based Continual Reassessment Method (CRM). This method uses registered primary outcome from the previous included patients or the prespecified prior if no data has been collected (see Fig. 1). The assigned methadone group is registered into REDcap and will be used to estimate the individual dose of methadone, which is calculated by multiplying methadone group with patient weight. This individual methadone dose will be written on a case report file (CRF). The primary care provider from the ED is responsible for withdrawing study medicine and placing the syringe near the patient together with the CRF. The individual dose of methadone is administered intravenously by the certified registered nurse anaesthetist when anaesthesia is commenced. The CRF will follow the patient to the orthopaedic ward.

## 7 Analysis

### 7.1 Descriptive statistics

We use and present descriptive statistics to ensure that the reader can evaluate if the sample is reproducible. For categorical variables numbers and percentages will be presented, and Fischer's exact or *χ*^2^-test will be used to assess if there is a difference between the groups defined by way of the exposure variables. For normally distributed variables, the mean and standard deviation will be presented, and ANOVA will be used to assess if there is a difference between the methadone dose groups. For non-categorical and non-normal distributed variables, median and interquartile ranges will be presented, and the Kruskal-Wallis test will be used to assess if there is a difference between groups.

### 7.2 Primary analysis and treatment assignment

This trial uses the Bayesian continual reassessment method, therefore stopping guidelines and probabilistic priors in relation to toxicity MTD (maximal tolerable dose) need to be determined before inclusion of the first patient. The stopping rules for the trial include:

• If 40 persons are included.
• If the probability of the lowest dose exceeding a predetermined toxicity threshold (set at 0.10) is greater than 95 % and more than 10 persons are included.
• If the 95 % credibility interval of the toxicity level for the MTD is between 0 and 0.10 (a clinically acceptable toxicity level) and more than 10 persons are included.
• If all the above stopping rules are fulfilled and 10 participants are included.

**Fig. 2:**
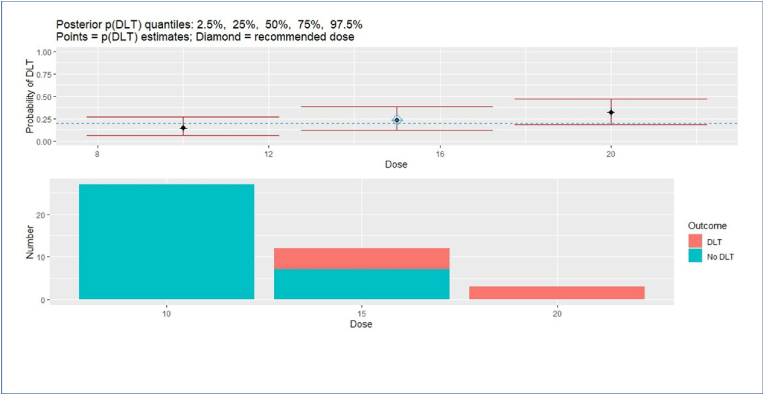
CRM output.

The predetermined toxicity threshold for the lowest dose considers the assumption that toxicity increases with higher doses. Thus, if the toxicity at the lowest dose surpasses the clinically acceptable level of 0.10, further dose escalation should be avoided. The pre-specified prior to this analysis consists only of presumed toxicity levels of the different doses and the alpha parameter. The presumed toxicity levels for the three doses is set to 0.05, 0.1, and 0.2 for 0.10 mg/kg, 0.15 mg/kg, and 0.2 mg/kg, respectively, and alpha follows a *γ*(1*,* 1) distribution. After the specification of the prior and stopping rules, the method uses the dose-response curve to escalate or deescalate the doses in a non-conservative manner (see Fig. 2) and close arms if one or more stopping rules are met. The 1-parameter model estimates the MTD more efficiently by escalating through the doses faster [16]. This is acceptable because the risk of adverse events by respiratory depression is low. Furthermore, there may be difficulties fitting the model or obtaining consistent estimates of model parameters in a two-parameter model [16]. Thus, we choose to use the 1-parameter model for this study.

### 7.3 Secondary analysis

The analysis of the secondary outcomes will consist of:

• Survival analysis focusing on the length of stay at the PACU. Kaplan Meier curves will be presented and pseudo-observations together with linear regression will be used to assess if the length of stay at the PACU differs between the three dose levels [17]. If the fit of the linear regression is not satisfactory, then bootstrapped confidence intervals and p-values will be presented instead of their parametric counterparts.
• Poisson regression will be used to investigate whether the number of times administration of Naloxone was necessary is dependent on the given dose level. The model control will be a graphical assessment of the deviance residuals. If the model control yields an unsatisfactory fit a negative binomial regression will be used instead.
• In relation to postoperative opioid consumption generalized estimating equations will be used as the variability is partially determined by the hospitals infrastructure, which is not reproducible. The p-value will be based on the contrast as time can be a potential effect modifier of the exposure. If convergence issues regarding the maximum likelihood estimate are present then a Poisson or negative binomial regression with clustered standard errors will be performed instead, depending on the distribution of the deviance residuals.
• Presence of opioid-related side-effects will be analysed by way of generalized estimating equations with a logit link function, as the variability is connected to the given hospital and therefore not reproducible. The p-value will be based on the contrast as time can be a potential effect modifier of the exposure. If convergence issues regarding the maximum likelihood estimate is present then a logistic regression with clustered standard errors will performed instead.

Postoperative pain will be analysed by way of a mixed effect model with bootstrapped confidence intervals, as the outcome is a Likert scale and the variability is fully determined by the given and is thus reproducible in other settings. The p-value will be based on the contrast as time can be a potential effect modifier of the exposure.

All the outcomes analysed by generalized linear mixed effect models or generalized estimating equations will be presented with margin plots to assess if there is a clinically significant difference between the groups. Conversely, if some effect measures yield a clinically insignificant difference between the exposure groups, strong predictors will be adjusted in the regression analysis to avoid bias for safety measures [18]. In order to avoid potential conservative bias as described by Möller et al. [18], clinically non-significant analyses will be conducted adjusted for strong predictors. The fundament for the evaluation of this bias is explicitly based on clinical expertise to eliminate the risk of analyses driven by statistical significance. In the literature e.g. a clinically relevant reduction in opioid consumption is set to 10 mg of morphine equivalent or a 30 % reduction [19].

### 7.4 Sample size calculation

To calculate a sufficient sample size for the primary analysis we choose the formula presented by Kuen Cheung et al. [20]. We set the target toxicity level to 0.10, an accuracy of 0.6 and an odds ratio to 2, as this is a fragile population. Therefore the project will include 40 patients unless the maximum tolerable dose meets the pre-specified stopping rules.

### 7.5 Reporting and interpretation of statistical measures

The adaptive dose-finding trial will use probabilistic methods. Therefore, 95 % credibility intervals will be reported in relation to the MTD and the prevalence of the given dose. In the secondary analysis, a p-value below 0.05 will be considered statistically significant, and 95 % confidence intervals will be reported together with their corresponding effect size. All p-values will be two-sided, and no adjustment for multiple testing will be utilized (as these analyses are not the primary analyses). All results will follow the Extended CONSORT guidelines for dose-finding studies if they are available [21].

### 7.6 Missing data

No missing data in relation to the primary analysis is expected as all measures are measured by healthcare professionals, therefore if data is missing it is assumed to be missing completely at random unless Little's test is statistically significant and no type I error seems to be present [22].

### 7.7 Time plan for final analyses and evaluation of effect measures

The statistical analysis will be conducted after the inclusion of the last patient and the publishing of this statistical analysis plan. The assessment of the effect measures will be conducted after the analyses have been performed.

### 7.8 Statistical software

The CRM will be conducted in R version 4.2.1 with the integrated development environment using the bcrm package [23] and the jags gibbs sampler. The rest of the analysis will be conducted in STATA ver. 17.

## 8 Discussion

The results of this study will provide important evidence on the analgesic safety measures of methadone during hip surgery. The maximal tolerable dose will be used in a future randomized clinical trial. This knowledge is essential when aiming to improve the treatment of hip-fracture patients. The study will run in a real-life setting to increase the feasibility of implementing the methods afterwards. This pre-defined SAP is essential to increase the study's transparency and explicitly describe protocol deviations to increase reproducibility and avoid any risk of reporting bias or data-driven analysis.

## Trial status

SAP version 1.0 was developed on the 15th of November 2022. Protocol to Ethics committee was updated on the 25th August 2022. Recruitment starts January 2023. Recruitment is estimated to be completed in June 2023.

## Steering commitee

This committee is composed of representatives from the participating departments: orthopedics, anesthesiology and research. The committee's role is to develop the scientific framework of the study and make final decisions on major issues during data collection and the data management period. Members of the steering committee are AKP, KHN, SRP, KSP, TS, CMM, HSA and JOS.

## Funding

Material costs are covered by grants from The A.P. Møller and Chastine Mc-Kinney Møller Foundation (grant number L-2022-00365) and Knud and Edith Eriksens memorial foundation (grant number 62786–2023). Investigator salary is covered by grants from The University Hospital of Southern Denmark (Kresten Philipsensvej 15, 6200 Aabenraa, Denmark;shs.kontakt@rsyd.dk) (grant number 22/25,256) and the Region of southern Denmark (grant number 22/26,251).

These financial sponsors have no influence on the data, analysis, results, or content of the publication.

## Availability of data and materials

Due to Danish laws on personal data, data cannot be shared publicly. The person responsible for the research is the principal investigator and corresponding author that together with the Department of Health Research and the University Hospital of Southern Denmark owns the data and has access to the final data-set. To request this data, please contact the corresponding author for more information. For ancillary studies, a new consent will need to be given by the Regional Committees on Health Research Ethics for Southern Denmark.

## Author's contributions

KHN, AKP, TS, KSP and JOS were involved in the planning of the study. KHN and AKP drafted the manuscript in collaboration with SRP. AKP and SRP contributed substantially with the statistical plan. KHN is the study investigator and JOS the research chief. All authors contributed with scientific knowledge. The work was revised by all authors, but especially HSA and CMM contributed significantly to the revision. The authors read and approved the final manuscript.

## Ethics approval

The project was approved by the National Committees on Health Research Ethics.

(S-20200133), registered by the Danish Data Protection Agency (22/29,376), by ClinicalTrials.gov (NCT05581901) and by the Danish Medicine Agency (2022063317).

## Consent to participate

Written informed consent will be obtained from the participants before participation in the study.

## Consent for publication

Not applicable.

## Declaration of competing interest

The authors declare that they have no known competing financial interests or personal relationships that could have appeared to influence the work reported in this paper.

## Data Availability

No data was used for the research described in the article.
